# Single-cell analysis reveals HBV-specific PD-1^+^CD8^+^ TRM cells in tumor borders are associated with HBV-related hepatic damage and fibrosis in HCC patients

**DOI:** 10.1186/s13046-023-02710-4

**Published:** 2023-06-23

**Authors:** Lulu Liu, Junwei Liu, Pan Li, Jijun Luo, Rui Qin, Qiao Peng, Bin Li, Xuyong Wei, Tian Wang, Hongyu Shi, Ming-Da Wang, Chao Li, Weijia Fang, Wei Chen, Xiao Xu, Tian Yang, Weiwei Yin, Xun Zeng

**Affiliations:** 1 Department of Medical Oncology, The First Affiliated Hospital, Zhejiang University School of Medicine, Hangzhou, 310000 China; 2 Key Laboratory for Biomedical Engineering of the Ministry of Education, College of Biomedical Engineering and Instrument Science, Zhejiang University, Hangzhou, 310058 China; 3 Department of Hepatobiliary and Pancreatic Surgery, Affiliated Hangzhou First People’s Hospital, Zhejiang University School of Medicine, Hangzhou, 310006 China; 4 Key Laboratory of Integrated Oncology and Intelligent Medicine of Zhejiang Province, Hangzhou, 310006 China; 5 Present Address: Guangzhou Laboratory, Guangzhou, 510005 Guangdong China; 6 State Key Laboratory for Diagnosis and Treatment of Infectious Diseases, National Clinical Research Center for Infectious Diseases, Collaborative Innovation Center for Diagnosis and Treatment of Infectious Diseases, The First Affiliated Hospital, Zhejiang University School of Medicine, Hangzhou, 310000 China; 7 Department of Thoracic Surgery, Zhejiang University School of Medicine, Sir Run Run Shaw Hospital, Hangzhou, 310016 China; 8 Department of Cardiology of the Second Affiliated Hospital, Zhejiang University, 866 Yuhangtang Road, Hangzhou, 310058 China; 9 School of Basic Medical Sciences, Zhejiang University, Hangzhou, 310058 China; 10 Department of Biological Testing, Zhejiang Puluoting Health Technology Co., Ltd, Hangzhou, 311121 China; 11 Department of Hepatobiliary Surgery, Eastern Hepatobiliary Surgery Hospital, Second Military Medical University (Navy Medical University), 225 Changhai Rd, Yangpu Qu, Shanghai, 200433 China; 12 Zhejiang University School of Medicine, Hangzhou, 310058 China; 13 Research Units of Infectious Disease and Microecology, Chinese Academy of Medical Sciences, Beijing, China

**Keywords:** Masscytometry, Hepatitis related liver disease, Tumor immunemicroenvironment, Virus specific T cells, Tissue tension

## Abstract

Immune checkpoint blockade (ICB) treatment of hepatocellular carcinoma (HCC) patients with hepatitis B virus (HBV) infection may activate viral-specific T cells to attack HBV infected hepatocytes and thus induce immune-related liver injury. Therefore, it is important to deeply understand the impacts of HBV infection on HCC immune microenvironment in order to better design effective immunotherapies for HBV^+^ (HBV infected) HCC patients. Here, We performed cytometry by time-of-flight (CyTOF) analyses to characterize the distinct immune compositions of HCC tumors, tumor borders, and their associations with HCC/HBV related clinical characteristics. We identified 31 distinct immune clusters and found significant associations between immune signatures with clinicopathological features of HCC. We further revealed the HBV infection had more effects on shaping immune compositions in tumor borders than in tumors, with the significant enrichment of HBV-specific PD-1^+^CD8^+^ tissue-resident memory T (T_RM_) cells in tumor borders of HBV^+^ patients. We confirmed this subset with a more exhausted phenotype and respond more actively under anti-PD-L1 treatment, suggesting its involvement in immune-related liver injury induced by ICB treatment to HBV^+^ HCC patients. Our study shows it may be necessary to consider antiviral prophylaxis for HBV^+^ HCC patients receiving ICB treatment.

**Supplementary Information:**

The online version contains supplementary material available at 10.1186/s13046-023-02710-4.

## Background

Hepatocellular carcinoma (HCC) accounts for 75% to 85% of the pathological types of primary liver cancer, and is the second most lethal tumor and the third leading cause of cancer-related deaths worldwide [1, 2]. Dysfunction of the immune surveillance, particularly in the local tumor immune microenvironment (TIME), is tightly associated with the occurrence and progression of HCC. Hepatitis B virus (HBV) is a widespread pathogen that is highly endemic in Southeast Asian countries [3]. Persistent HBV infection could induce liver damage and lead to persistent cell death, compensatory regeneration, and liver fibrosis, part of which finally induces HCC [4]. By comparing the intratumoral immune components, HBV^+^ (HBV infected) HCC patients had a more exhausted and immunosuppressive microenvironment than HBV^−^ (non-HBV infected) HCC patients [5]. However, it is still unclear how the HBV-induced immunosuppression affects the cellular immune components within the TIME and what are the clinical benefits of immune checkpoint blockades (ICB) treatment for HBV^+^ HCC patients [6]. A recent study of immunotherapy trials in HCC claimed a comparable objective response rate between HBV^+^ and HBV^−^ patients, but lower disease control rates in HBV^+^ patients [7]. Moreover, since HBV antigens can be expressed on tumoral hepatocytes, HBV-specific T cells can eliminate tumoral hepatocytes by recognizing HBV antigens and are thus regarded as potential immunotherapeutic targets for treating HBV^+^ HCC. However, non-tumoral hepatocytes can also express HBV antigens and thus be potentially attacked by HBV-specific T cells and lead to immune-mediated liver injury [8]. Therefore, it raises the safety concerns whether ICB treatment can activate HBV-specific T cells to massively attack HBV-infected non-tumoral hepatocytes and thus induce liver injury. Indeed, ICB treatment can elevate alanine aminotransferase/aspartate aminotransferase (ALT/AST) levels, a signature of hepatic damage, and potentially cause progressing hepatic failure [9]. Moreover, compared with HCC patients without virus infection, Nivolumab (anti-PD-1) treatment potentially increased higher levels of the serum ALT/AST in HCC patients with HBV/HCV virus infection, implying that ICB treatment could damage more hepatocytes in viral infected HCC patients [10]. Therefore, it is urgent to know the exact characteristics of the HBV-induced immunosuppressive microenvironment in tumors and normal liver tissues, especially the precise role of HBV-specific T cells in HBV^+^ HCC. All of that would facilitate the better design of ICB treatment to precisely activate immune cells and to avoid massive lysis of non-tumoral hepatocytes.

Here, we applied single-cell cytometry by time-of-flight (CyTOF) analyses to reveal the distinct immune signatures between liver tumors and tumor borders. We compared the immune compositions between HBV^+^ and HBV^−^ HCC patients and found that PD-1^+^CD8^+^ tissue-resident memory T (T_RM_) cells were more highly associated with HBV infection, hepatic damage and fibrosis in tumor borders rather than in tumors. Besides, these cells had more immunosuppressive phenotypes, and were highly enriched with HBV-specific T cells. Comparing to HBV antigen stimulation alone, these cells exhibited more activated state under HBV antigen and anti-PD-L1 treatment. All together, our data identified HBV-related immune alteration in HCC patients and suggested that the immunosuppression in tumor borders due to HBV infection might contribute to liver damage and fibrosis, raising more concerns about ICB treatments probably involved with viral-specific T cell responses to non-tumoral hepatocytes for HBV^+^ HCC patients.

## Materials and methods

### Patients and specimens

Fresh liver lesions for CyTOF analyses were respectively collected from 30 patients diagnosed with HCC, and patients with concurrences of autoimmune disease, HCV, HIV, and syphilis were excluded. For HCC patients, tissues from HCC tumor (referred as INT) and tumor border (referred as TB) were collected based on their histological features. The location of the tumor border was defined as 2 cm away from the paired tumor tissues. And 25 INT and 25 TB specimens were finally resected for CyTOF analysis. The clinical characteristics of HCC patients for CyTOF analysis were summarized in Tables S1, S2 and S3. Further paired sets of INT and TB samples for external validation were collected from 29 HCC patients. The clinical characteristics of HCC patients enrolled for external validation were summarized in Table S6. All patients received no treatment against cancer (including anti-tumor drug treatment or radiotherapy) before surgery and written informed consent was obtained from each patient, and the study protocol was approved by the Ethics Committee of the first affiliated hospital, Zhejiang University School of Medicine (Hangzhou, China).

### Single-cell suspension preparation

Fresh tissues were digested to generate single-cell suspensions as previously described [11]. Briefly, the resected tissues were washed by PBS and minced, then subjected to the enzymatic digestion mix (collagenase IV (Sigma, V900893), deoxyribonuclease type I (Sigma, D5025), and hyaluronidase type V (Sigma, H3506)). The mixed suspension was shaken for 60 min at 37 °C for full digestion and then filtered through the 70-mesh filter screen. After washed with Cell Staining Buffer (CSB, PBS containing 0.5% BSA and 0.02% NaN_3_), the cell pellet was resuspended into ACK lysing buffer to remove the red blood cells. The rest of the single cells were conducted by using the percoll density gradient media (GE Healthcare) to get rid of fats and debris. The single-cell suspensions in CSB were then collected for CyTOF and flow cytometry analyses.

### Antibody staining for CyTOF analyses

An immune cell-centric antibody panel that includes 42 antibodies was listed in Table S4. For cell staining, single-cell suspension was resuspended in PBS and incubated with 1 μM Cisplatin (Fluidigm) at room temperature for 5 min. After washing with CSB, cells were blocked with blocking buffer (10% normal mouse serum, 10% normal human serum in CSB) for 20 min on ice, followed by staining with cell-surface antibodies on ice for 30 min. For intracellular markers staining, cells were fixed with 2% of PFA and incubated with DNA Intercalator-Ir (Fluidigm) at 4 °C for overnight, and then permeabilized using the Foxp3/Transcription factor staining buffer (eBiosciences) followed by intracellular antibodies staining for 30 min on ice. After that, cells were washed and diluted with EQ normalization beads containing ^140^Ce, ^151^Eu, ^153^Eu, ^165^Ho, and ^175^Lu (Fluidigm) and introduced into the CyTOF system (Helios, Fluidigm). FCS files were uploaded for downstream analysis.

### Multiplex Immunofluorescence Staining

Formalin-fixed, paraffin-embedded (FFPE) tissue sections of INT and TB tissues were obtained from the patient cohort who underwent CyTOF analysis. Slides were deparaffinized and rehydrated by using xylene and a graded ethanol series. For antigen retrieval, the slides were treated with 3% H_2_O_2_ for 15 min and heated in citrate buffer until boiling. The slides were stained with the primary antibodies diluted in 1% BSA in PBS overnight at 4 °C, followed by incubating slides in the appropriate secondary antibody diluted in 1% BSA in PBS for 1 h at room temperature and then were washed and mounted in Vectashield mounting medium with DAPI (Vector Laboratories). Sections were imaged using a high-resolution digital Axio Scan.Z1 slide scanner and associated Zen software (Olympus FV3000). Primary and secondary antibodies purchased from Abcam as follows: mouse anti-human PD-1 (NAT105), rabbit anti-human CD103 (EPR22590-27), rat anti-human CD8 (YTC182.20), Alexa Fluor 647-conjugated Goat anti-rat IgG H&L preadsorbed, Alexa Fluor 594-conjugated Goat anti-rabbit IgG H&L preadsorbed, Alexa Fluor 488-conjugated Goat anti-mouse IgG H&L preadsorbed.

### HLA protein refolding, biotinylation, and tetramer preparation

Peptides of HLA-A*02:01-restricted HBV-derived epitopes (core 18–27 (FLPSDFFPFV), envelope 183–191 (FLLTRILTI), envelope 335–343 (WLSLLVPFV), polymerase 455–463 (GLSRYVARL), and polymerase 502–510 (KLHLYSHPI)) were purchased from Genescript, China, and dissolved in DMSO (Sigma). HLA protein was refolded and biotinylated as described [12]. To prepare the tetramer, biotinylated HLA-peptide mixed with PE-streptavidin (Biolegend) at the molar ratio of 4:1 on ice for 30 min.

### Flow cytometry and cell sorting

All the antibodies for flow cytometry were purchased from BioLegend unless otherwise stated. Single cells from paired INT and TB tissues were pre-incubated with blocking buffer (TruStain FcX from Biolegend) for 20 min on ice, followed by stained with antibody cocktails for another 30 min on ice. The following antibodies, coupled to the appropriate fluorochromes, were used: anti-human CD3ε (OKT3), CD4 (A161A1), CD45 (HI30), CD19 (SJ25C1), CD11b (ICRF44), CD8 (DAKO; DK25), PD-1 (A17188B), CD103 (Ber-ACT8), CD14 (HCD14), CD56 (5.1H11), CD137 (4B4-1), IFNγ (4S.B3). life/dead Zombie Aqua™ Fixable Viability Kit (Biolegend) were used as viability dyes. For tetramer staining, PE-conjugated streptavidin-HLA-peptide tetramer were used. For cell sorting, Auqa^−^CD45^+^CD19^−^CD14^−^CD56^−^CD11b^−^CD3^+^CD4^−^CD8^+^CD103^+^ and PD-1^+^ cells were sorted into CSB for RNA isolation. For intracellular staining, the Golgi Plug protein transport inhibitor brefeldin A (BioLegend) was added for the last 4 h of stimulation. Cells were fixed and permeabilized with Cytofix/Cytoperm solution (BD) and stained with antibodies against intracellular antigens for 30 min on ice. Flow Cytometry was performed on BD LSRFortessa or CytoFLEX and DxFLEX flow cytometer (BECKMAN COULTER). Cell sorting was performed on Sony SH800. Data were analyzed using FlowJo v.10 software.

### HBV peptide pool stimulation

A HBV peptide pool containing 360 synthetic peptides, 15 amino acids in length with 11 overlapping amino acids covering the entire amino acid sequence of HBV genotypes B and C, with purity of more than 95%, was used as antigens (Genescript). Peptides were dissolved in DMSO and aliquots were stored at -80℃. Single cell suspensions extracted from tumor borders were cultured in AIM-V medium (Thermofisher) supplemented with Penicillin/Streptomycin/glutamine (Thermofisher) at 1 × 10^7^ cells/ml in a 96 well U-bottom plate. Cells were stimulated with 15-mer overlapping peptide pools for 24 h and DMSO was used as a negative control. To evaluate the function of the HBV-specific T cells, Single cell suspensions extracted from tumor borders were stimulated with HBV peptide pool in the presence or absence of 10 μg/ml anti-human PD-L1 antibody (durvalumab) for 24 h.

### Bulk RNA-Seq

Target immune cell populations were sorted from the paired INT and TB tissues of HBV^+^ and HBV^−^ HCC patients. The total RNA of sorted PD-1^+^ T_RM_ cells (CD8^+^CD103^+^PD-1^+^) were isolated using a Picopure RNA-Isolation kit (Arcturus, Ambion) according to the manufacturer's instructions. cDNA was generated using SMART Seqv4 UltraLow Input RNA Kit (Clontech), Illumina-ready libraries were prepared from cDNA using the Illumina-NexteraXT DNA-Library-Prep Kit (Illumina). Sequencing was performed at the NGS Platform of Biomarker Technologies Company on a Novaseq6000 platform using S4 Reagent Kit V1.0.

### Pre-processing and analysis of CyTOF data

The raw fcs files were normalized by bead standards [13], and debarcoded by the sample-specific channel staining [14]. After that, fcs files were imported into FlowJo (10.0.7) software for manually gating single and live CD45^+^ cells. In order to remove the effects of Gadolinium contamination induced by the usage of Gadolinium (Gd)-based contrast reagent in MRI scanning before tissue resection, pre-selected non-expressed markers in Gd isotope channels for major immune cell subtype were used to filter out the severely contaminated single cells. The raw counts of single cells were transformed by arcsinh with a factor of 5, and the self-organized map neural network [15] was used to cluster single cells. A default 10 × 10 network was used for over-clustering, and then manually merged into biological meaningful immune subsets based on hierarchical clustering and expert experience for downstream analysis. And the data were visualized by the tSNE projections [16].

### Ishak scoring

The specimens obtained from surgical resection were fixed, paraffin-embedded, and stained with hematoxylin–eosin. Appropriate diagnosis of a specimen included the observation of at least six portal areas in the sample. To avoid differences and the bias between examiners, all data were examined and evaluated by a single experienced pathologist blinded to the clinical data. Necroinflammation and fibrosis were scored using the Ishak scoring system [17].

### Transcriptome data analysis

The quantification of transcript expressions was performed by using the ‘quant’ function in the ‘kallisto’ software with the reference of GRCh38 [18]. The transcript abundance files were imported by the ‘tximport’ R package, and the transcript expressions were converted into the gene expressions by using the ‘EnsDb.Hsapiens.v86’ R package. For downstream analysis, the rarely expressed genes were filtered by the ‘filterByExpr’ function, the normalized gene expressions were used for PCA projections and heatmap visualization. For analysis of differentially expressed genes (DEGs) across sample groups, the ‘limma’ R package was used [19], the DEGs were defined as abs(*p*.value) < 0.05 and abs(logFC) > 1. And the pathway analysis was performed by using the ‘gost’ function in the ‘gprofiler2’ R packages [20].

### Obtaining and analysis of the scRNA-seq dataset

The validating scRNA-seq datasets were downloaded with the ID (GSE140228). The gene-cell expression matrix and the information of single cells were obtained, and we then subset T cells using the ‘Lymphoid-T’ label in the celltype_global feature and ‘Normal’ and ‘Tumor’ labels in the Tissue feature. The gene-cell expression matrix of T cells was split into objects and import into the Seurat data analysis workflow for data normalization, integration, and clustering analyses [21]. Using the clustering resolution of 1, we obtained 17 T cell clusters. we then used the ‘FindAllMarkers’ to identify the DEGs of each T cell cluster for identifying the T_RM_ cells. To further compare the functional modifications of T_RM_ cells within different tissues and HBV infections, we classified T cells in the identified cell clusters into 4 T cell groups and compared the DEGs of HBV^+^ and HBV^−^ T_RM_ cells within different tissues using the function ‘FindMarkers’. The AUCell scores of the expression of different gene signature in single cells were calculated with the function “AUCell calcAUC” in the “AUCell” package. The pathway analysis was performed as described before.

### Statistical analyses

The unpaired student’s t-test was used to compare the frequency differences across sample groups, and Spearman's correlation test was used in correlation analyses. Two-way ANOVA was used to compare the changes of major immune cell frequencies across HBV infection and tissue sites. All tests were performed as two-tailed tests, and for all tests, significance levels were defined as *, *P* < 0.05; **, *P* < 0.01; ***, *P* < 0.001; and ****, *P* < 0.0001.

## Results

### High-dimensional immune-cell profiling of HCC microenvironment

**Fig. 1:**
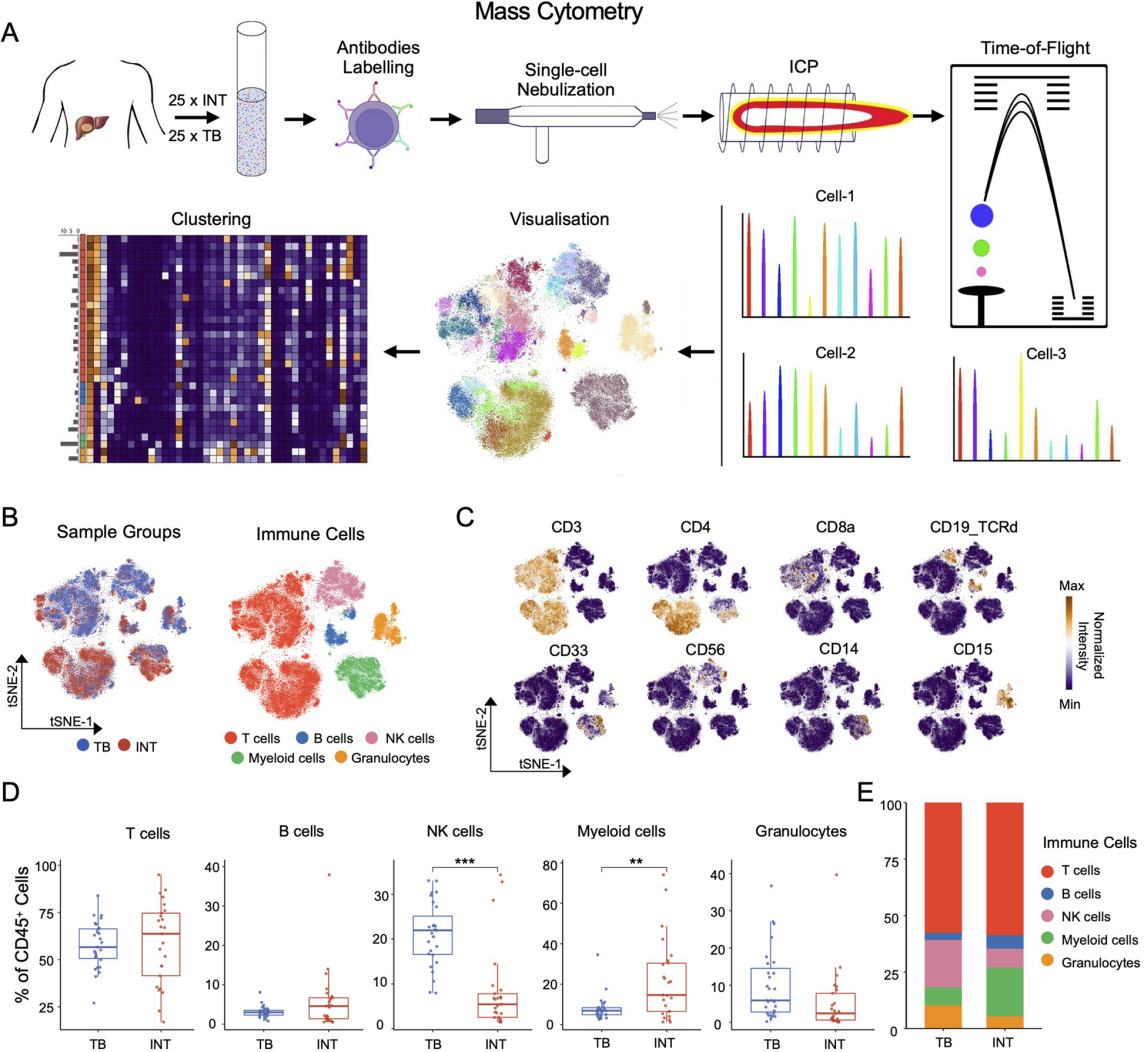
Distinct immune components across HCC tissues. **A** The schematic diagram of CyTOF experiments in this study. **B** The tSNE plots of immune cells from all samples, colored by different tissues (left) and immune cell types (right). **C** The expressions of selected markers on tSNE plots as in (B), the CD19_TCRd marker means sharing detection channels of CD19 and TCRd markers, with mutually independent expressed on B cells and γδ T cells. **D** Frequency comparisons of major immune cell subsets across tissue sites. **E** The barplot shows the compositions of the major immune components in different tissue sites. Unpaired student’s t-test was used in (**D**), with *p < 0.05, **p < 0.01, and ***p < 0.001

To characterize the immune compositions in HCC microenvironment at a single-cell level (Fig. 1A), we performed CyTOF analyses on freshly resected tissues from 30 treatment-naïve HCC patients. Tissues from HCC tumor (referred as INT, *n* = 25) and tumor border (referred as TB, *n* = 25) were separately collected from the enrolled cohort. Detailed sample information and patients’ clinical characteristics of the CyTOF cohort were listed in Table S1 and Table S2.

The CyTOF data were collected, pre-processed, and normalized, and we obtained 5,322,457 single cells in total (Figure S1A). For clustering analysis, we applied a self-organizing map (SOM) [15] to separate immune cells into 100 subclusters according to the designated immune marker panel (Figures S1B and C). We then classified subclusters into 5 major immune subsets including T cells, B cells, NK cells, Myeloid cells, and Granulocytes based on the expression of immune lineage markers (Figs. 1B and C). By comparing the major immune components across tissues, we found that the frequencies of NK cells and myeloid cells were respectively increased (*p* < 0.001) and decreased (*p* < 0.01) in TB compared to INT, whereas the frequencies of other types of cells were similar in both tissues (Figs. 1D and E).

### Distinct immune components of INT and TB tissues in HCC

**Fig. 2:**
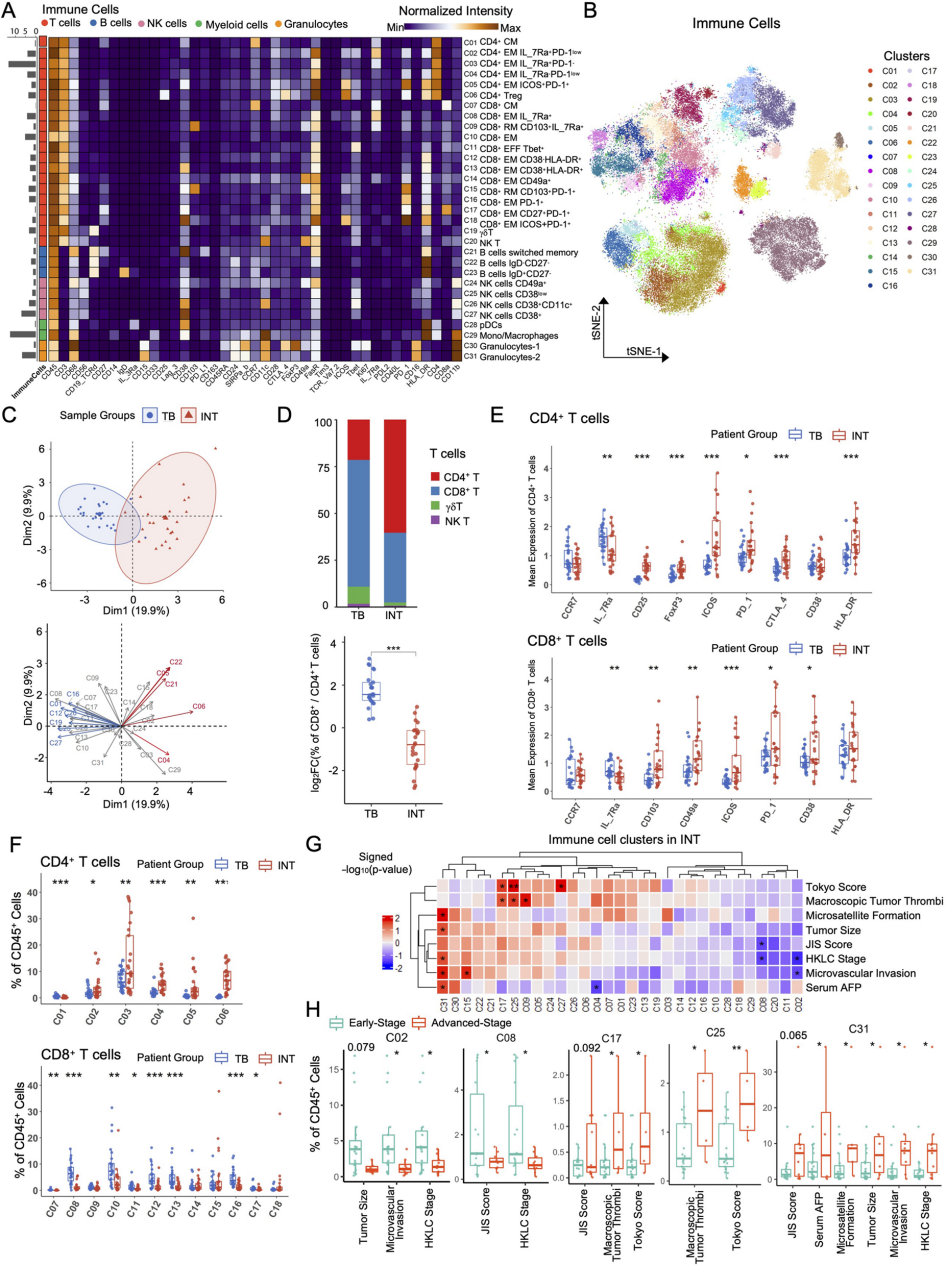
The detailed immune components across HCC tissues. **A** The heatmap of meta-clusters of immune cells from all samples, colored by major cell subsets and labeled with cluster frequencies on the left, detailed cluster annotation was labeled on the right. **B** The tSNE plot of immune cells from all samples, colored by meta-clusters. **C** The PCA projections of immune signatures for different tissue samples, colored by tissue sites (top). The important immune features are labeled with colored arrows (bottom). **D** The barplot shows the compositions of major T cell subsets for different tissue sites (top); Frequency comparisons of the ratio of CD4^+^/CD8^+^ T cells across tissue sites (bottom). **E** Mean expression comparisons of selected markers in CD4^+^ (top) and CD8^+^ (bottom) T cells between INT and TB tissues. **F** Frequency comparisons of CD4^+^ (top) and CD8.^+^ (bottom) T cell clusters between INT and TB tissues. **G** The heatmap shows the significance level of frequency comparison for meta-cluster across patients grouped by defined clinical features in INT tissues, colored by the signed -log_10_(*p*-value). **H** Frequency comparisons for meta-clusters in INT tissues across patients grouped by the defined clinical features. HCC patients were divided into early-stage and advanced-stage groups according to the level of corresponding clinical parameters listed in supplemental table 5. Unpaired student’s t-test was used in (**D**-**H**), with *p < 0.05, **p < 0.01, and ***p < 0.001

To depict the detailed immune landscape across tissues, the clustered 100 subclusters were meta-clustered into 31 biological meaningful immune clusters, including 20 T cell clusters (C01-C20), 3 B cell clusters (C21-C23), 4 NK cell clusters (C24-C27), 2 Myeloid cell clusters (C28-C29), and 2 Granulocyte clusters (C30-C31) (Figs. 2A and B). We applied Principal Component Analysis (PCA) to compare the immune composition variability between individual samples and tissue groups. The generated 2D projection showed rare overlaps across tissue groups, suggesting the heterogeneous immune compositions between INT and TB (Fig. 2C).

T cells are crucial to eliminate malignant cells and the dysfunctional states of infiltrated T cells (inactivated or exhausted) are directly associated with tumor progression [22]. We classified T cells into 4 lineage T cell subsets (CD4^+^ T, CD8^+^ T, γδ T, and NK T cells), among which CD8^+^ T cells and γδ T cells were more abundant in TB than that in INT, and CD4^+^ T cells were the dominant T cell subset in INT. Further analysis revealed the lower ratio of CD8^+^ to CD4^+^ T cells in INT than that in TB, suggesting fewer cytotoxic cells existing inside tumors than at tumor border (Fig. 2D).

To further characterize the distinct phenotypes of T cells between INT and TB groups, we compared the mean expression of selected functional markers respectively for CD4^+^ and CD8^+^ T cells. Compared to TB, T cells in INT expressed higher levels of PD-1 and ICOS for both CD4^+^ and CD8^+^ T cells, higher expressions of CD25, Foxp3 on CD4^+^, and higher expressions of CD103, CD49a on CD8^+^ T cells, respectively (Fig. 2E). Comparing the abundances of identified CD4^+^ (C01–C06) and CD8^+^ T cell clusters (C07–C18) between two tissues, we revealed CD4^+^ effector memory T cells (CD4^+^ T_EM_, C02-C05) and Tregs (C06) were highly enriched in INT with PD-1 expression on most of CD4^+^ T cell clusters (Figs. 2A and F). As for CD8^+^ T cell clusters, most of them were more abundant in TB. Furthermore, similar to most NK cells, γδ T cells and NK T cells (C19, C20) were more enriched in TB than that in INT, indicating the infiltration and accumulation of these cells specifically in tumor border tissues (Figure S2A).

### The associations between clinical outcomes and immune features in HCC

As clinicopathological features are pivotal indicators for clinical and progression diagnosis of HCC [23], we explored the relationship between HCC-related clinical features/scores and our identified CyTOF immune signatures. We collected and calculated several widely used clinicopathological features and scores according to different staging systems [24], including serum AFP (Alpha-Fetoprotein), tumor size, macroscopic tumor thrombi, microvascular invasion, microsatellite formation, Tokyo score, JIS (Japan Integrated Staging) score, and HKLC (Hong Kong Liver Cancer) stage. Based on these clinical criteria, the CyTOF cohort patients were classified into either the early-stage or advanced-stage groups. The detailed clinical information, the classification criteria, and the number/percentage of patients in each stage group were summarized in Table S2 and S5.

We compared the immune distributions between early-stage and advanced-stage groups in INT and TB tissues respectively and summarized the results by signed transformed *p*-values with a positive value reflecting immune cluster enrichments in the advanced-stage group, and vice versa (Fig. 2G and Figure S2B). We found that PD-1^+^CD27^+^CD8^+^ T_EM_ cell (C17), CD38^low^ NK cell (C25), and granulocytes-2 (C31) cell clusters were significantly enriched in the advanced-stage group, while PD-1^low^IL-7Ra^+^CD4^+^ T_EM_ cells (C02) and IL-7Ra^+^CD8^+^ T_EM_ cells (C08) in INT were more enriched in the early-stage HCC patients (Figs. 2G and H). These patterns were only observed inside tumors, but not at tumor borders. We also inspected the associations between immune signatures identified in TB with the clinical features and revealed a higher abundance of Tregs (C06), PD-1^+^ICOS^+^CD8^+^ T_EM_ cells (C18), CD49a^+^ NK cells (C24) in tumor border for advanced HCC patients (Figure S2B and C). All of these analyses demonstrate the distinct association between specific immune subsets in local TIME and global phenotypic disease progression as indicated by the clinicopathological features/scores, suggesting these immune signatures could potentially be used for clinical evaluation after tumor resection.

### HBV infection affects immune compositions in HCC tissues

**Fig. 3:**
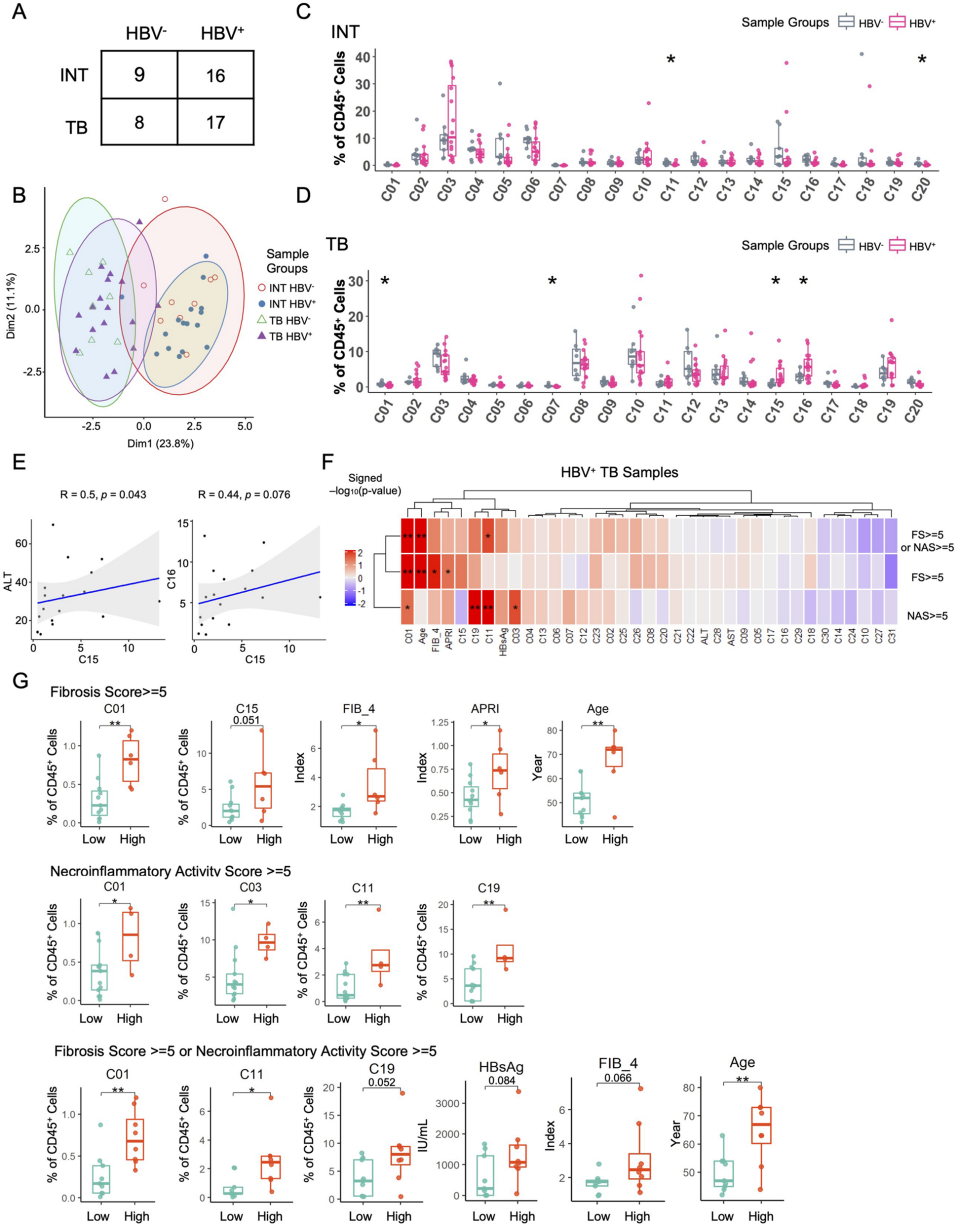
The HBV-related immune alteration in INT and TB tissues. **A** The number of samples in patient groups used for identification of HBV-related immune alterations. **B** The PCA projections of immune signatures for all samples, colored by different sample groups. **C** and **D** Frequency comparisons of the meta-clusters between HBV^+^ and HBV^−^ patients in INT tissues (**C**) and TB tissues (**D**). **E** The correlation plots between the frequency of C15 and ALT (left), and the frequency of C16 (right) in TB tissues of HBV^+^ patients. **F** The heatmap shows the significant level of frequency comparison for meta-cluster across TB tissues of HBV^+^ patients grouped by different histopathological scores, colored by the signed -log_10_(*p*-value). **G** Frequency comparisons of the meta-cluster in TB tissues of HBV^+^ patients grouped by different histopathological scores. HBV infected HCC patients were divided into low-grade or high-grade liver disease based on criteria using Fibrosis Score and/or Necroinflammatory Activity Score as listed in supplemental table 7. Unpaired student’s t-test was used in (**C**, **D**, **F**, and **G**), with *p < 0.05, **p < 0.01, and ***p < 0.001. Spearman correlation was used in E, the correlation coefficients and *p*-value was labeled

As HBV infection is one of the major risk factors in the pathogenesis of HCC [23], we then classified the CyTOF cohort patients into HBV^+^ and HBV^−^ groups based on the patients’ HBV serological markers and HBV DNA levels [25] and obtained four groups based on HBV infection and tissue site (Fig. 3A). We performed PCA analyses and found a clear tissue site-dependent separation of these groups. Two INT groups mostly overlapped with each other regardless of the HBV infection status (Fig. 3B). And the situation in the two TB groups was similar (Fig. 3B), indicating that tumorigenesis had a greater impact on the overall variance of immune signatures than HBV infection. We subsequently compared the major immune compositions across four groups and found that the different patterns of major immune subsets were more related to tissue sites but not HBV infection (Figure S3A). By comparing the detailed immune compositions, we found that in INT tissues only two minor subsets with low abundance (C11: CD8^+^ effector T cells, C20: NK-T cells) were significantly different between HBV^+^ and HBV^−^ groups. While, there were more subsets (C01: CD4^+^ T_CM_ cells, C07: CD8^+^ T_CM_ cells, C15: PD-1^+^CD8^+^ T_RM_ cells, and C16: PD-1^+^CD8^+^ T_EM_ cells) significantly changed in TB tissues under different HBV status, suggesting that HBV infections have more impact on immune signatures in TB tissues (Figs. 3C and D and Figures S3B and C).

To further investigate how immune clusters in TB tissues are related to HBV infections, we analyzed the correlations between TB immune signatures and HBV-related clinical features within HBV^+^ patients (*n* = 21) from the CyTOF cohort (Table S2). We collected three HBV serological markers (Hepatitis B surface antigen ‘HBsAg’, Hepatitis B e antigen ‘HBeAg’, Anti-Hepatitis B e antigen ‘Anti-Hbe’), the HBV DNA level, two biochemical parameters (AST/ALT), two noninvasive fibrosis markers (aspartate aminotransferase-to-platelet ratio index ‘APRI’, Fibrosis-4 index ‘FIB-4’), and two histopathological scores (Fibrosis score ‘FS’, Necroinflammatory activity score ‘NAS’), and used these HBV and liver disease clinical markers to represent the grade of necroinflammation and the stage of fibrosis caused by chronic HBV infection [25]. The detailed HBV-related clinical features, corresponding classification thresholds, and distribution of these HBV^+^ patients were summarized Table S2 and S6.

The correlation analyses revealed many significantly correlated pairs between the frequency of individual immune subsets and the aforementioned HBV-related clinical features (Figure S3D). CD4^+^ T_CM_ cells (C01), CD8^+^ T_CM_ cells (C07), and IgD^+^CD27^−^ naive B cells (C23) were significantly and positively correlated with the level of HBsAg; IL-7Ra^+^PD-1^−^CD4^+^ T_EM_ cells (C03), CD38^+^HLA-DR^+^CD8^+^ T_EM_ cells (C13), and γδ T cells (C19) were positively correlated with necroinflammatory activity score; IL-7Ra^+^CD8^+^ T_EM_ cells (C08) and memory B cells (C21) were positively correlated with the level of noninvasive fibrosis marker FIB-4 (Figures S3D and E). For the two immune clusters (C15 and C16) that significantly increased in TB tissues due to HBV infection, we found that they were potentially correlated with each other and further identified C15 was positively correlated (*p*-value = 0.043) with the ALT level, an important indicator for hepatic damage of inflammation. This result suggests the potential role of C15 at tumor border in immune attack during HBV infection (Fig. 3E).

We further divided these HBV^+^ patients from the CyTOF cohort into two groups, the low-grade and high-grade groups of liver disease based on their fibrosis scores and necroinflammatory activity scores or both scores[17] that were the morphological features used for staging liver chronic hepatitis (Table S7). We then compared the distribution of TB immune clusters between these two groups (Fig. 3F). The high-grade group is considered using three different criteria with either FS or NAS equal or greater than 5, or both. We revealed highly enrichment of CD4^+^ T_CM_ cells (C01) in the high-grade group for all three criteria, and also a higher frequency of PD-1^+^CD8^+^ T_RM_ cells (C15) in the advanced fibrosis group using the criterion FS equal or greater than 5 (Fig. 3G). We found immune clusters C03, C11, and C19 significantly increased in the severe necroinflammation group when the criterion of NAS was used, suggesting their potential roles in HBV-induced hepatic necroinflammation (Figs. 3F and G).

### PD-1^+^CD8^+^ TRM cells were enriched in TB tissues of HBV^+^ HCC patients

**Fig. 4:**
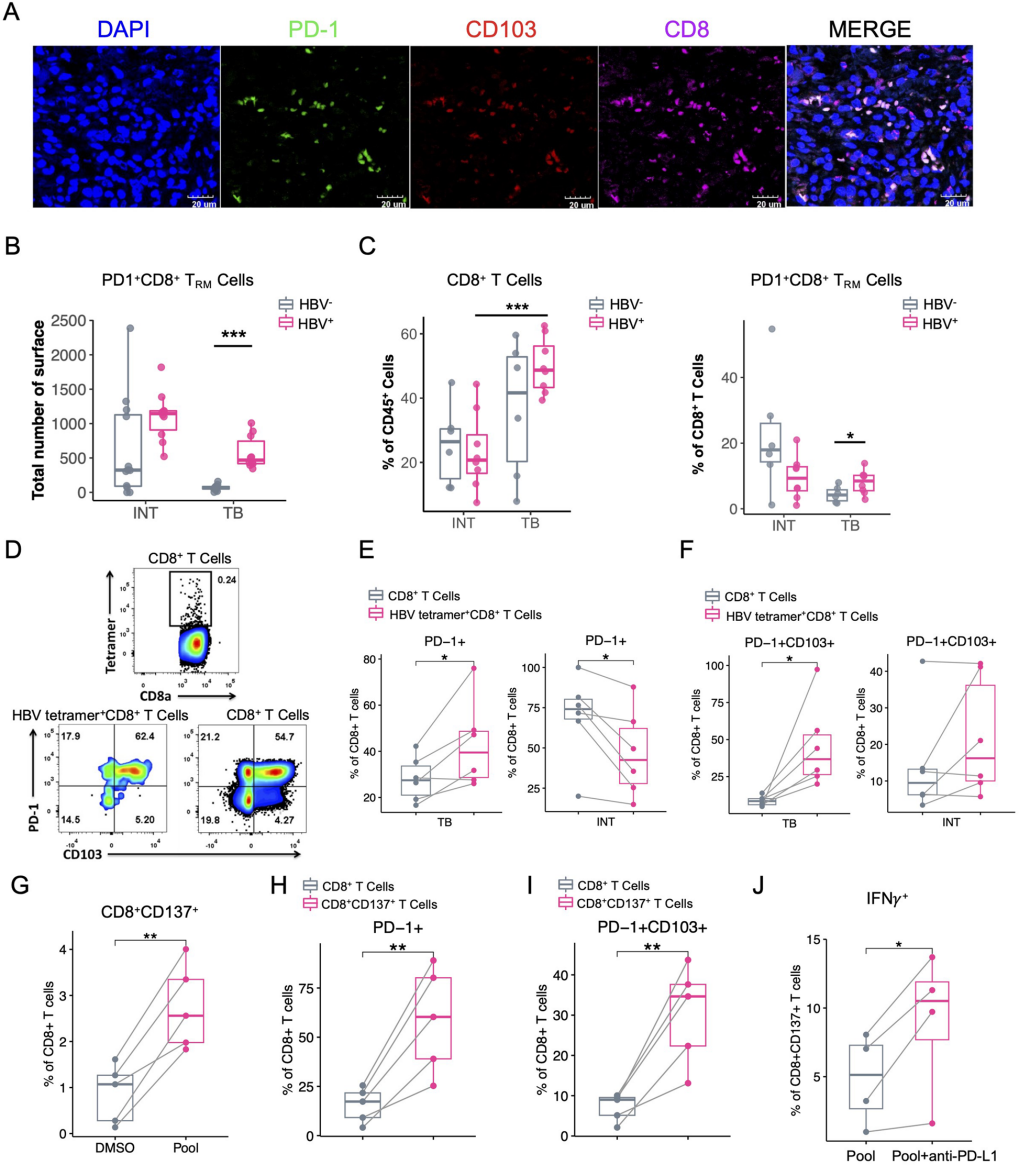
Validation of the existence of PD-1^+^CD8^+^ T_RM_ cells in HBV^+^ samples. **A** Multiplex immunofluorescence staining of formalin-fixed paraffin-embedded TB tissues of enrolled HCC patients in CyTOF analyses, stained with different markers, and the colocalized cells were colored by white, scale bar represents 20 μm. **B** Comparisons of the relative counts of PD-1^+^CD8^+^ T_RM_ cells across sample groups by multiplex immunofluorescence images as in (**A**). **C** Frequency comparisons of CD8^+^ T cells in CD45^+^ cells (left) and PD-1^+^CD8^+^T_RM_ cells in CD8^+^ T cells (right) of different sample groups, colored by HBV infections. **D** The representative flow cytometry plots of HBV tetramer pool and surface marker staining and gating strategies of different cell subsets. **E** and **F** Frequency comparisons of PD-1^+^ T cells in CD8^+^ T cells and HBV tetramer^+^CD8^+^ T cells (**E**) and PD-1^+^CD8^+^ T_RM_ cells in PD-1^+^CD8^+^ T cells and PD-1^+^HBV tetramer^+^CD8^+^ T cells (**F**). **G** Frequency comparison of CD8^+^CD137^+^ T cells in CD8^+^ T cells stimulated with DMSO or HBV-specific antigen peptide pools, colored by treatment groups. **H** and **I** Frequency comparison of PD-1^+^ T cells (**H**) and PD-1^+^CD8^+^T_RM_ cells (**I**) in paired CD8^+^ and CD8^+^CD137^+^ T cells of HBV-specific antigen peptide pools stimulated T cells. **J** Frequency comparison of IFNγ^+^CD8^+^ T cells in stimulated CD137^+^CD8.^+^ T cells treated with HBV-specific antigen peptide pools in the presence or absence of of anti-PD-L1 mAb. Unpaired student’s t-test was used in (**B**, **C**, **E**, and **F**), Paired t-test was used in (G-J), with *p < 0.05, **p < 0.01, and ***p < 0.001

Since PD-1^+^CD8^+^ T_RM_ cells (C15) responded to HBV infection, we then applied multiplexed tissue immunofluorescence to validate the existence of these cells. Using the samples from the CyTOF cohort, we found that the number of PD-1^+^CD8^+^ T_RM_ cells in TB was significantly higher in the HBV^+^ group compared to HBV^−^ group, and this phenomenon did not exist in INT, both of which were well consistent with our CyTOF results (Figs. 4A and B, Figure S4A).

We further verified these results using an extra HCC validation cohort by flow cytometry (*n* = 14), with 8 patients diagnosed as HBV^+^ HCC and 6 patients diagnosed as HBV^−^ HCC (Table S3). Comparing the distinct immune cell frequencies between both groups, we revealed the frequency of total CD8^+^ T cells was highly increased only in TB for HBV^+^ group, but not in INT tissues. Similarly, the PD-1^+^CD8^+^ T_RM_ cells were also significantly enriched in TB but not in INT tissues as well. These results confirmed the specific enrichment of CD8^+^ T cells, particularly PD-1^+^CD8^+^ T_RM_ cells in TB tissues for HBV^+^ HCC patients (Fig. 4C).

Next, we asked whether these T_RM_ cells were HBV-specific. We then selected 6 HBV^+^ patients with HLA-A*02:01 background from the above flow cytometry validation cohort and stained the INT and TB samples from these patients with a combined HLA-A2-HBV peptide tetramer pool (Fig. 4D). The patients' HBV and HCC-related clinical information of the external validation cohort were summarized in Table S3. We revealed a higher proportion of PD-1^+^ and PD-1^+^CD103^+^ CD8^+^ T cells in HBV tetramer^+^CD8^+^ T cells than that in total CD8^+^ T cells in TB, but not in INT (Fig. 4E), suggesting that HBV-specific CD8^+^ T cells are more exhausted and enriched in PD-1^+^CD8^+^ T_RM_ cells in TB (Fig. 4F).

Since the HBV peptide tetramer staining can only present part of HBV-specifc T cells, we used a HBV overlapping peptide pool to stimulate single cell suspensions extracted from tumor borders derived from HBV^+^ patients to evaluate a more comprehensive functional characterization of HBV-specific T cells in TIME [26]. The expression of CD137, an activation-induced surface receptor [27], was upregulated in CD8^+^ T cells, supporting the existence of HBV-specific CD8^+^ T cells in TIME (Fig. 4G). We then compared the PD-1 expression in these HBV-specific T cells, and found that ~ 60% of CD8^+^CD137^+^ T cells expressed PD-1, and ~ 40% of them were PD-1^+^CD8^+^ T_RM_ cells, consistent with tetramer staining data (Fig. 4H, I). Next, we compared the expression of IFNγ in CD8^+^ T cells in TB with TCR-stimulations by a HBV peptide pool combined with anti-PD-L1 treatment or not. The frequency of IFNγ^+^CD8^+^ T cells was increased in anti-PD-L1 treated group, proposing ICB treatment could more efficiently activate HBV-specific CD8^+^ T cells when they were stimulated by HBV antigens (Fig. 4J). Taken together, these data confirm that HBV-specific T cells were enriched in PD-1^+^CD8^+^ T_RM_ cells in HBV^+^ TB tissues and ICB treatment induced the better activation of HBV-specific T cells.

### Transcriptional heterogeneity of PD-1^+^CD8^+^ TRM cells isolated from HBV^+^ and HBV^−^ HCC tissues

**Fig. 5:**
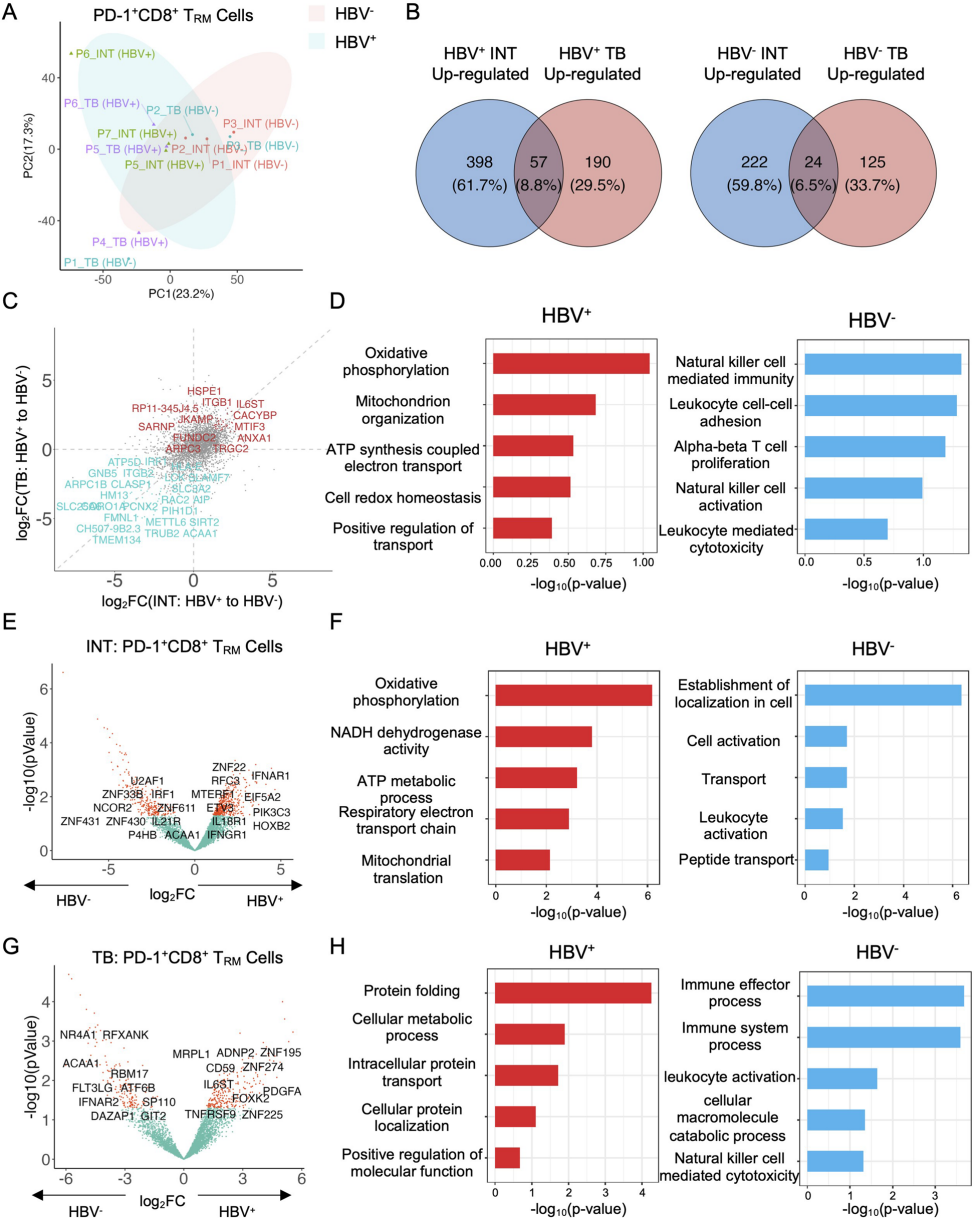
Transcriptomics profiling of PD-1^+^CD8^+^ T_RM_ cells across HBV infection and tissue sites. **A** The PCA projections of the transcriptomic profiling of sorted PD-1^+^CD8^+^ T_RM_ cells for all samples, colored by different sample groups, and ellipses indicate differences between HBV^+^ and HBV^−^ samples. **B** The Venn plots of shared upregulated (left) and downregulated (right) differentially expressed genes of PD-1^+^CD8^+^ T_RM_ cells between HBV^+^ and HBV^−^ samples within INT and TB tissues. **C** The scatter plot of the log_2_(fold change) of genes between HBV^+^ and HBV^−^ samples within INT and TB tissues, colored and labeled by shared upregulated and downregulated genes. **D** The GO pathway analyses of shared upregulated genes (left) and downregulated genes (right) between HBV^+^ and HBV^−^ samples as in (**C**), the pathway names were labeled. **E** and **G** The volcano plots of differentially expressed genes between HBV^+^ and HBV^−^ samples in INT tissues (**E**) and TB tissues (**G**), labeled with typical marker genes. **F** and **H** The GO pathway analyses of shared upregulated genes (left) and downregulated genes (right) between HBV^+^ and HBV^−^ samples in INT tissues (**F**) and TB tissues (**H**), the pathway names were labeled

To further identify the functionalities of these T cells, we performed RNA-seq analyses to characterize the transcriptional heterogeneities of PD-1^+^CD8^+^ T_RM_ cells isolated from HCC tissues with or without HBV infection. And we sorted CD45^+^CD19^−^CD11b^−^CD3^+^CD4^−^PD-1^+^CD103^+^CD8^+^ T cells from the freshly resected INT and TB tissues from the HCC validation cohort (*n* = 6) for downstream analyses (including 3 HBV^+^ HCC patients and 3 HBV^−^ HCC patients, Table S3). The PCA projection of all samples indicated that the HBV infection contributed more to the variance of RNA-seq samples compared to the tissue site, suggesting HBV infection as one of the major factors affecting the functionalities of these CD8^+^ T_RM_ cells (Fig. 5A). To identify the different transcriptomic phenotypes of these cells, we compared the differentially expressed genes (DEGs) of PD-1^+^CD8^+^ T_RM_ cells between HBV^+^ and HBV^−^ HCC patients within INT or TB or both tissues, respectively. Interestingly, we revealed 57 upregulated genes (8.8% of total identified genes) and 24 downregulated genes (6.5%) were shared between different tissue sites (Figs. 5B and C). By performing Gene Ontology (GO) pathway enrichment analysis on these shared genes in both INT and TB tissues, we found in HBV^+^ HCC samples, the up-regulated DEGs of PD-1^+^CD8^+^ T_RM_ cells were enriched in oxidative phosphorylation (OXPHOS) related pathways, whereas in HBV^−^ HCC patient the up-regulated DEGs were enriched in immune activation pathways (Fig. 5D and Figure S5A). Separately comparing the DEGs between HBV^+^ and HBV^−^ samples in INT and TB tissues (Figs. 5E-H and Figures S5B and C), we found the PD-1^+^CD8^+^ T_RM_ cells exhibited more activated and effector phenotypes in HBV^−^ HCC patients in both INT and TB tissues. In contrast, as for the HBV^+^ HCC patients, these cells in INT and TB tissues were enriched in cell metabolic pathways.

### Functional differences in TRM cells isolated from HBV^+^ and HBV^−^ HCC tissues

To further characterize the functional phenotypes of PD-1^+^CD8^+^ T_RM_ cells in HBV-associated HCC tissues, we re-analyzed single-cell RNA sequencing (scRNA-seq) datasets from a published study by *Zhang *et al. [28]. Their study enrolled two HCC patients without HBV infection (HBV^−^) and three HCC patients under HBV infections (HBV^+^), and all tumor (INT) and adjacent liver (TB) tissues of these patients were profiled with scRNA-seq. We subset the annotated ‘Lymphoid-T’ cells and cells in ‘Tumor’ and ‘Normal’ tissues from their datasets, and finally obtained 17,810 T cells for downstream analyses.

**Fig. 6:**
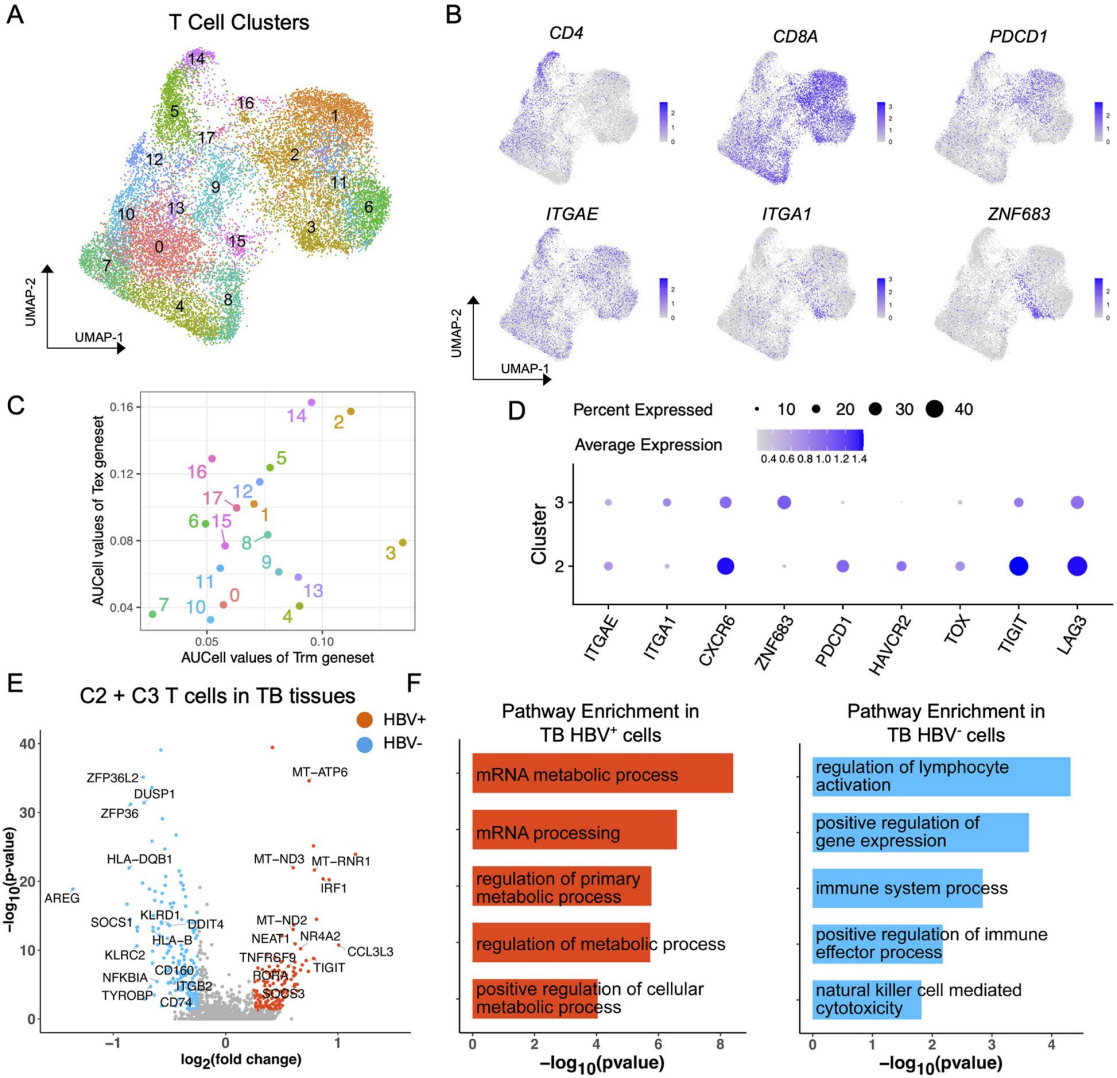
Analysis of validating scRNA-seq datasets. **A** The UMAP plot of subset T cells, colored by T cell clusters. **B** The UMAP plots of the expression of selected genes as in (**A**). **C** The scatter plot of the AUCell expressions of gene signatures of exhausted T cells and tissue-resident memory T cells between T cell clusters, colored by cell clusters. **D** The dot plot of the expression percentage and intensity of genes in exhausted T cells and tissue-resident memory T cells between C2 and C3 T cell clusters. (**E**) The volcano plots of differentially expressed genes of C2 + C3 T cells between HBV + and HBV- samples in TB tissues, labeled with typical marker genes. **F** The GO pathway analyses of shared upregulated genes (left) and downregulated genes (right) between HBV + and HBV- C2 + C3 T cells in TB tissues, the pathway names were labeled

The single-cell clustering analysis of these T cells identified 18 T cell clusters (Fig. 6A, Figure S6A), and the result of differentially expressed genes of T cell clusters showed the both C2 and C3 T cell clusters mostly resembled T_RM_ cells with expressions of typical T_RM_ markers *ZNF683* and *ITGA1*[29, 30] (Fig. 6B). To identify T cell clusters most similar to the C15 T cell cluster in CyTOF result, we calculated the mean expression of T cell clusters in typical gene signatures of tissue-resident memory T cells (T_RM_: *ITGAE*, *ITGA1*, *CXCR6*, *ZNF683*) and exhausted T cells (Tex: *PDCD1*, *HAVCR2*, *TOX*, *TIGIT*, *LAG3*, *NR4A1*) (Fig. 6C). Both C2 and C3 clusters showed the T_RM_ and Tex signatures, although the C2 T cell cluster showed lower expression of T_RM_ gene signature compared to C3 cluster but a high expression of Tex gene signature, along with higher expressions of *PDCD1*, *HAVCR2*, *TOX*, *TIGIT*, and *LAG3* (Fig. 6D). Considering that our flow cytometry and CyTOF data had limited markers to define T_RM_ cells that may include both C2 and C3 clusters, we combined C2 and C3 clusters together as T_RM_ cells for further analysis. We compared the functions of these CD8^+^ T_RM_ cells (C2 + C3) within different tissues and HBV infection status. In INT tissues, we identified the higher expressions of exhaustion-related genes (*PDCD1*, *HAVCR2*, *TIGIT*) in CD8^+^ T_RM_ cells from HBV^+^ patients (Figure S6B). GO enriched pathway analysis revealed that CD8^+^ T_RM_ cells in both HBV^+^ and HBV^−^ INT tissues were immune reactive (Figure S6C). However, in TB tissues, we identified high expression of NK cell related cytotoxic genes (*KLRD1*, *KLRC2*), and higher enrichment of immune response and T cell killing pathway on T_RM_ cells in HBV^−^ patients (Figs. 6F). Our results indicated that CD8^+^ T_RM_ cells had impaired immune responses in HBV^+^ TB tissues, consistent with our RNA-seq data, which suggests the HBV infection may contribute to immune suppression in HCC tissues.

## Discussion

The tumor heterogeneity not only affects the progression of tumors but also the efficacies of anti-tumor therapies [31], among which the reprogramming of its local TIME during tumorigenesis would be a major factor resulting ineffective immunosurveillance, therapeutic failures, and worse patient prognosis [32]. Our results demonstrated distinct immune signatures in different HCC liver tissue sites. The identifications of few CD8^+^ T cells and abundant Treg cells in INT tissues and enriched NK cells and CD8^+^ T cells in TB tissues confirmed again the more immune suppressive microenvironment in the tumor core with a more cytotoxic border around it.

We further compared the relevance of these immune signatures with clinical outcomes, granulocytes-2 (C31) in INT tissues enriched in multiple clinical outcomes with poor prognosis and was identified as potential risk factors of HCC. Granulocytes alone or neutrophil-to-lymphocyte ratio (NLR) in peripheral blood were reported to associate with the poor prognosis in HCC [33, 34], and infiltrated neutrophils could recruit macrophages and Treg cells to promote immunosuppressive TIME contributing to tumor progression in HCC [35]. On the other hand, IL-7 had antitumor activity by improving T cell cytotoxic and noncytotoxic activity in HCC [36]. Therefore, it is not surprising that the two highest IL-7Ra expressing T cell clusters, PD-1^low^IL-7Ra^+^CD4^+^ T_EM_ cells (C02) and IL-7Ra^+^CD8^+^ T_EM_ cells (C08), might act as antitumor long-live memory T cells [37, 38]. The findings of these immune signatures and their potential linkages to clinicopathological features could benefit HCC phenotypes classification in the future.

HBV infection can induce immune suppression and increase the risk of HCC incidents [39]. The impact of HBV infection in the liver immune microenvironment has been comprehensively investigated [40, 41]. *Lim *et al*.* revealed the enrichment of Treg cells and CD8^+^ T_RM_ cells in HBV^+^ INT tissues and suggested the abundance of these cells connected to the prognosis of HCC patients [5]. Our study confirms their Tregs findings, and beyond that, we revealed that the PD-1^+^CD8^+^ T_RM_ cells (C15) were enriched in TB tissues rather than INT tissues, particularly in HBV^+^ HCC patients. The differences between both studies might due to the complex immune regulations in INT tissues with both malignant cells and HBV infection. In addition, we further confirmed these cells were HBV-specific by additional HBV peptide tetramer pool staining and ex vivo stimuation [42, 43]. Moreover, upon correlation analysis with a set of clinical features, we found that the enrichment of PD-1^+^CD8^+^ T_RM_ cells (C15) in TB tissues was tightly associated with hepatic damage and fibrosis, and this is the first time to show T_RM_ cells were correlated with HBV-associated fibrosis.

During chronic HBV infection, the majority of PD-1^+^CD8^+^ T_RM_ cells in the liver were HBV specific and could release IFNγ and IL-2 with HBV antigen stimulation [36]. We confirmed that PD-1^+^CD8^+^ T_RM_ cells were not correlated with serum HBsAg level [36], but correlated with serum ALT level, suggesting tumor progression may promote PD-1^+^CD8^+^ T_RM_ cells to damage normal tissues as non-tumoral hepatocytes can also express HBV antigens and became the targets of HBV-specific T cells. We revealed that HBV-specific T cells are CD103^+^ T_RM_ cells with higher expression of PD-1 and respond actively with more IFNγ release under ICB treatment by a HBV peptide pool stimulation ex vivo. On the other hand, by using RNA-seq, we revealed that the HBV infection significantly induced the immune-suppressive phenotypes of PD-1^+^CD8^+^ T_RM_ cells in both INT and TB tissues. Unexpectedly, we found the OXPHOS-related pathways were enriched in PD-1^+^CD8^+^ T_RM_ cells in HBV^+^ INT tissues, which was distinct from HBV-specific CD8^+^ T cells in the chronic HBV infection [44, 45]. Our data implied that these PD-1^+^CD8^+^ T_RM_ cells in HBV^+^ tissues had relatively normal metabolic function with less T cell activation compared to the HBV^−^ counterparts.

In conclusion, we revealed the enrichment of PD-1^+^CD8^+^ TRM cells in HBV^+^ TB tissues with HBV specificity associated with hepatic damage and fibrosis. Our finding suggested that HBV-induced PD-1^+^CD8^+^ T_RM_ cells may potentially aggravate tissue lesions which may further facilitate tumor progression. Particularly, our results would shed light on a deeper understanding of hepatic adverse events encountered during ICB treatment which was more common in HBV infection patients [46]. We therefore raise the necessity of antiviral prophylaxis for HBV infection patients receiving ICB treatment, which has important clinical significance for improving ICB treatment safety.

## Supplementary Information

**Additional file 1:** **Supplemental table 1.** Summary of the resectedtissues from HCC patients.

**Additional file 2:** **Supplemental table 2.** The clinicalcharacteristics of enrolled patients for CyToF analysis.

**Additional file 3:** **Supplemental table 3.** HBV associatedclinicopathological features in HBV+ group of CyTOF pateints cohort.

**Additional file 4:** **Supplemental table 4.** Antibody staining panel forCyTOF experiments.

**Additional file 5:** **Supplemental table 5.** HCC associated clinicalcharacteristics of CyTOF analysis cohort.

**Additional file 6:** **Supplemental table 6.** The clinicalcharacteristics of enrolled patients for validation experiments.

**Additional file 7:** **Supplemental table 7.** Classification of HBV+patients with TB tissues based on the grade of liver disease.

**Additional file 8:** **FigureS1.**CyTOF data analyses of immune cells from HCC and HH tissues.(A) Thegating strategy of CyTOF data for live and singleton immune cells. (B) The tSNEplots of selected markers as in (Figure 1B). (C) The heatmap of 100 SOMsubclusters, major immune cell subtypes, and cluster frequencies were labeledon the top.

**Additional file 9:** **FigureS2.** The clinical-related immunealterations in TB tissues. (A) Comparisons of the frequencies of selectedmeta-clusters between INT and TB tissues. (B) The heatmap of the comparisons ofmeta-cluster frequencies across patients grouped by defined clinical featuresin TB tissues, colored by the signed -log_10_(p-value). (C)Comparisons of the meta-cluster frequencies in TB tissues between early-stageand advanced-stage patients classified as in (B). Unpaired student’s t-test wasused in (A-C), with *p < 0.05, **p < 0.01, and ***p < 0.001.

**Additional file 10:** **FigureS3.**Correlation of HBV clinical features with immune features. (A) Comparisons ofthe frequencies of major immune subsets across HBV infection and HCC tissues.(B and C) Comparisons of the frequencies of selected meta-clusters between HBV+and HBV- samples in INT (B) and TB (C) tissues. (D) The heatmap of the Spearmancorrelation coefficients between HBV clinical features and immune features inTB tissues, colored by correlation coefficients and for pairs with *p*-value morethan 0.1 were set to zero. E, The correlation plots between selected HBVclinical features and immune features as in (D). Two-way ANOVA test was used in(A), with *p < 0.05, **p < 0.01, and ***p < 0.001.

**Additional file 11:** **FigureS4.**Validation of the existence of PD-1^+^CD8^+^ T_RM_cells in HBV^+^ samples. (A) Multiplex immunofluorescence staining offormalin-fixed paraffin-embedded INT and TB tissues of enrolled HCC patients as shown in Figure 4A.

**Additional file 12:** **Figure S5. **The distinct geneexpression of PD-1^+^CD8^+^ T_RM_ cells across HBVinfection and tissue sites. (A) The heatmap of the normalized expressions ofshared upregulated and downregulated genes between HBV^+^ and HBV^-^samples in INT and TB tissues, color-labeled by HBV infection. (B and C) Theheatmap of the normalized expressions of selected functional genes of enrichedGO pathways between HBV^+^ and HBV^-^ samples in INT tissues(B) and TB tissues (C), color-labeled by HBV infections.

**Additional file 13:** **FigureS6.** Analysis results ofscRNA-seq datasets of HCC T cells. (A) The UMAP plots of T cells in HCCpatients as in (Figure 6A), colored by patient ID (left), tissues (middle), andHBV infection status (right). (B)The volcano plots of differentially expressed genes of C2+C3 T cells between HBV^+^and HBV^-^ samples in INTtissues, labeled with typical marker genes. (C) The GO pathway analyses of shared upregulatedgenes (left) and downregulated genes (right) between HBV^+^ and HBV^-^C2+C3 T cells in INT tissues, the pathway nameswere labeled.

## Data Availability

The datasets generated during the current study are available from the corresponding author upon reasonable request.

## Abbreviations

ICB: Immune checkpoint blockade
HCC: Hepatocellular carcinoma
HBV: Hepatitis B virus
CyTOF: Cytometry by time-of-flight
TRM: Tissue-resident memory T
TIME: Tumor immune microenvironment
ALT/AST: Alanine aminotransferase/aspartate aminotransferase
SOM: Self-organizing map
