# Supplementary figures and images for: Single-cell analysis reveals HBV-specific PD-1+CD8+ TRM cells in tumor borders are associated with HBV-related hepatic damage and fibrosis in HCC patients
Source: J Exp Clin Cancer Res. 2023 Jun 23;42:152. doi: 10.1186/s13046-023-02710-4 (PMC10288678; doi:10.1186/s13046-023-02710-4)

Figure S1

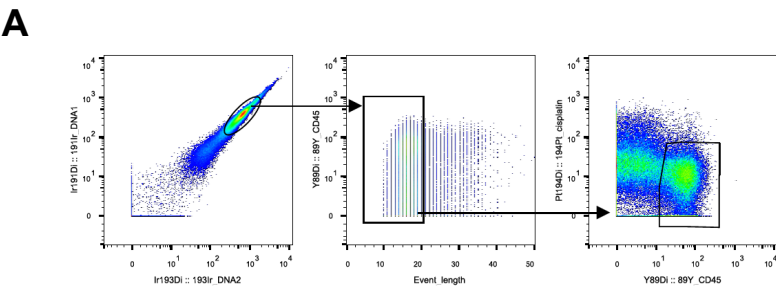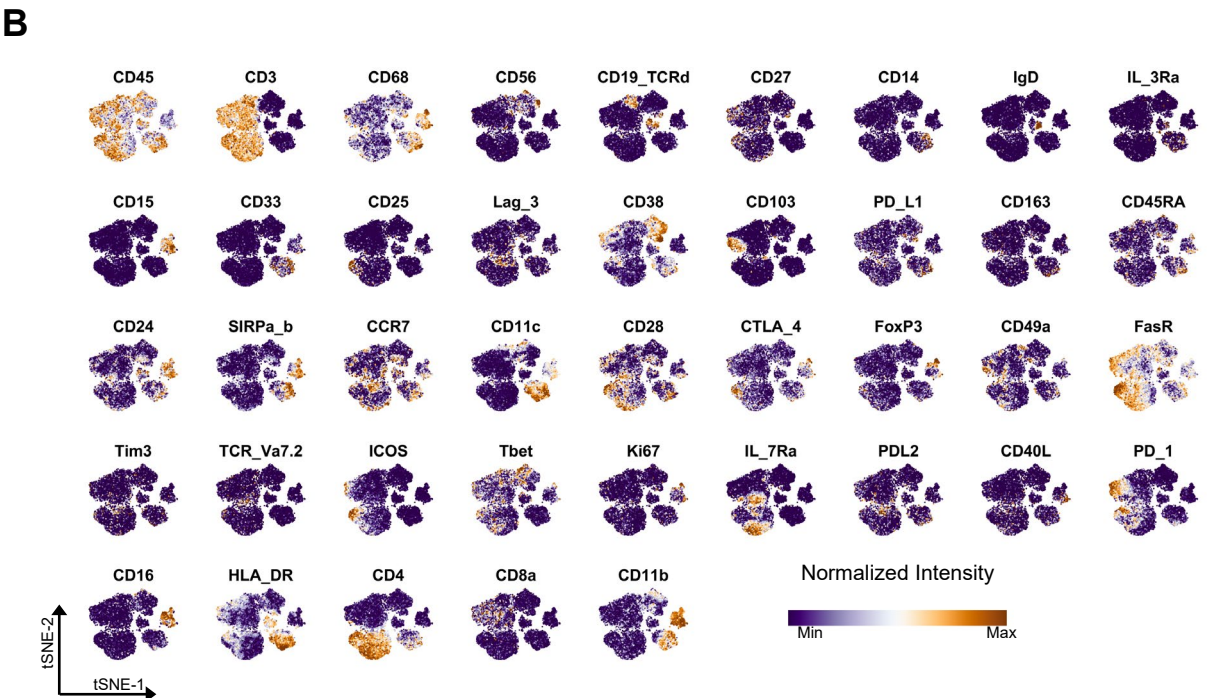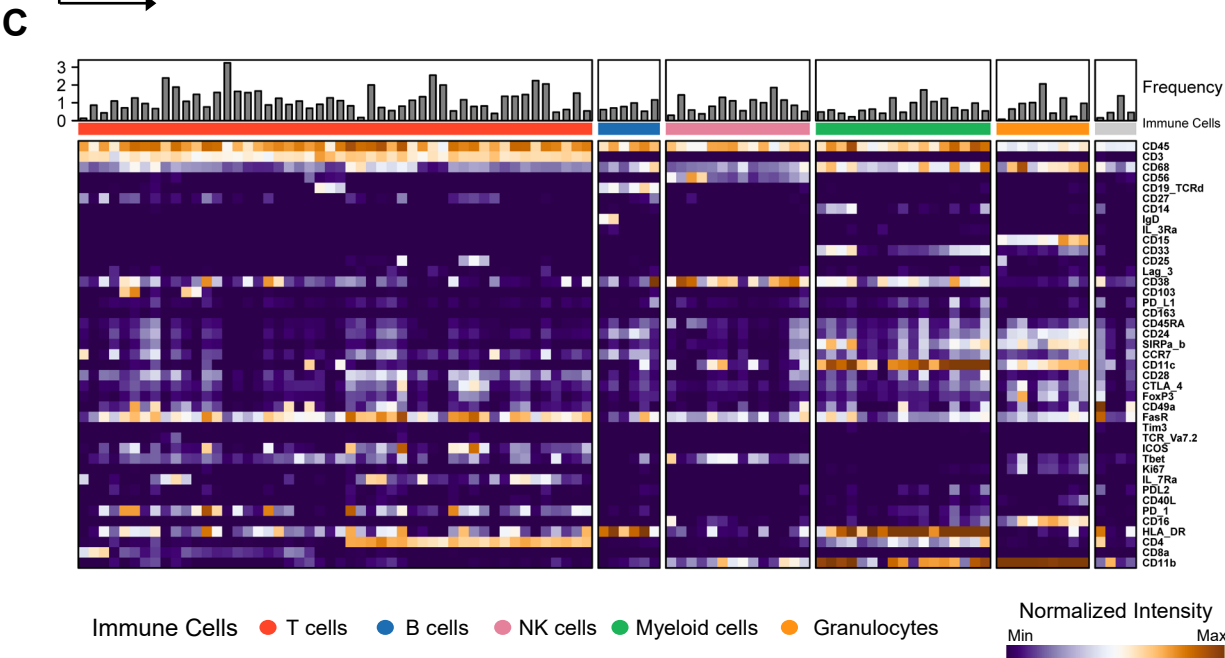

Supplement: Supplementary file 8 — Additional file 8: FigureS1.CyTOF data analyses of immune cells from HCC and HH tissues.(A) Thegating strategy of CyTOF data for live and singleton immune cells. (B) The tSNEplots of selected markers as in (Figure 1B). (C) The heatmap of 100 SOMsubclusters, major immune cell subtypes, and cluster frequencies were labeledon the top. [file 13046_2023_2710_MOESM8_ESM.pdf]

Figure S2

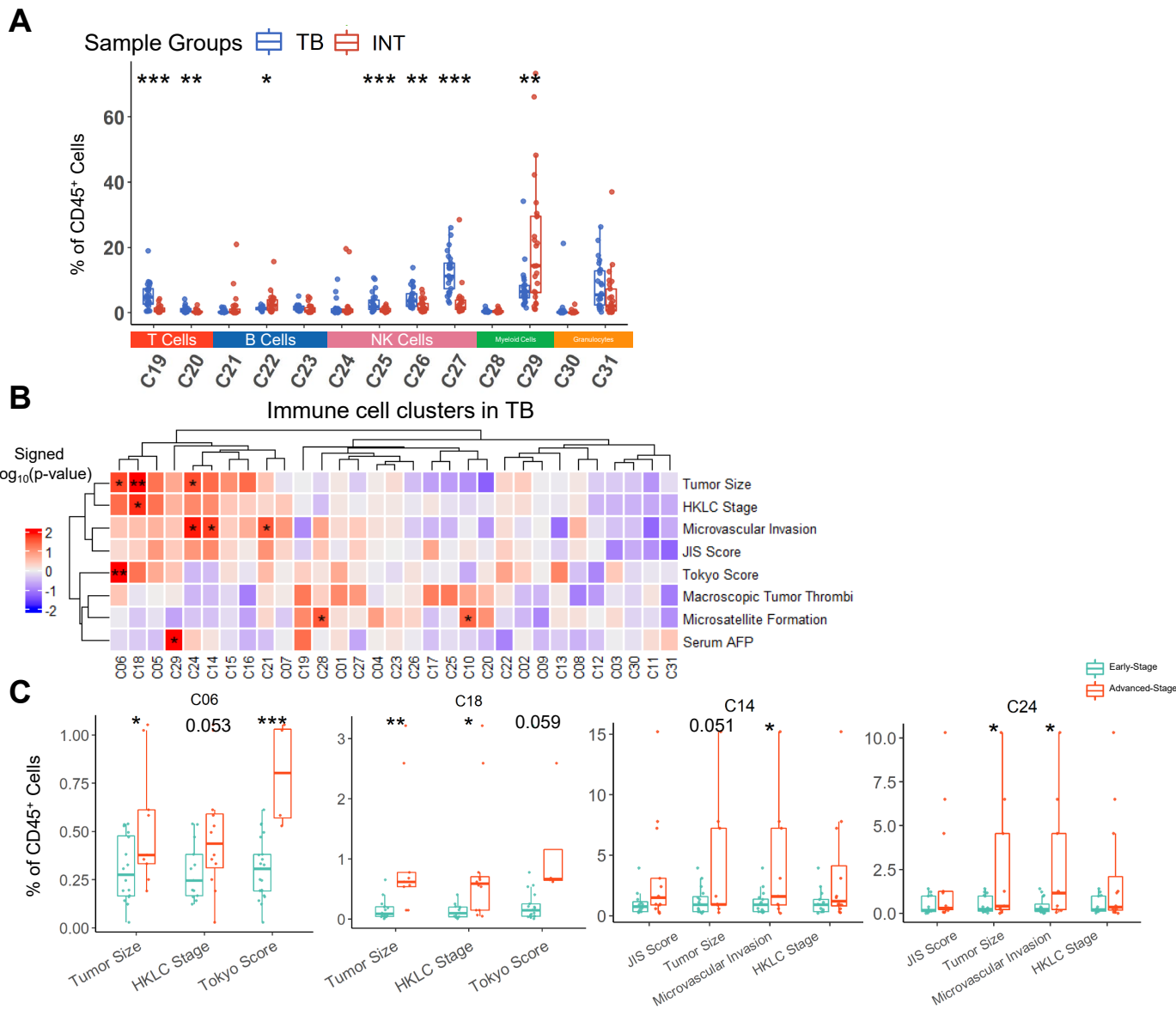

Supplement: Supplementary file 9 — Additional file 9: FigureS2. The clinical-related immunealterations in TB tissues. (A) Comparisons of the frequencies of selectedmeta-clusters between INT and TB tissues. (B) The heatmap of the comparisons ofmeta-cluster frequencies across patients grouped by defined clinical featuresin TB tissues, colored by the signed -log10(p-value). (C)Comparisons of the meta-cluster frequencies in TB tissues between early-stageand advanced-stage patients classified as in (B). Unpaired student’s t-test wasused in (A-C), with *p < 0.05, **p < 0.01, and ***p < 0.001. [file 13046_2023_2710_MOESM9_ESM.pdf]

Figure S3

A

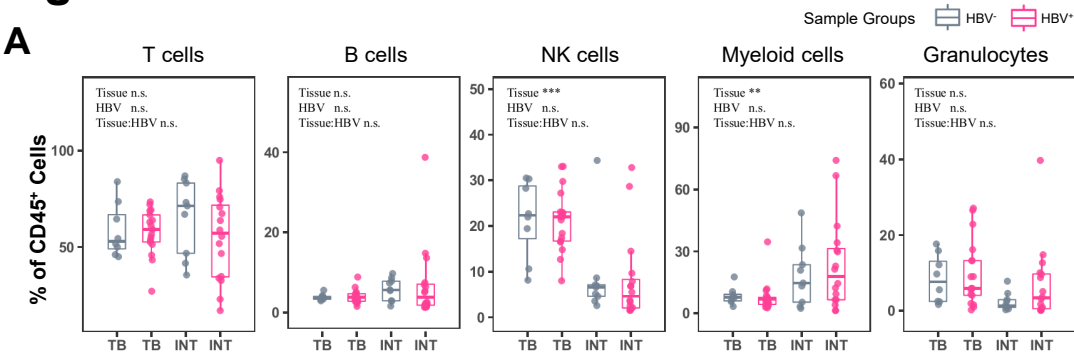

B

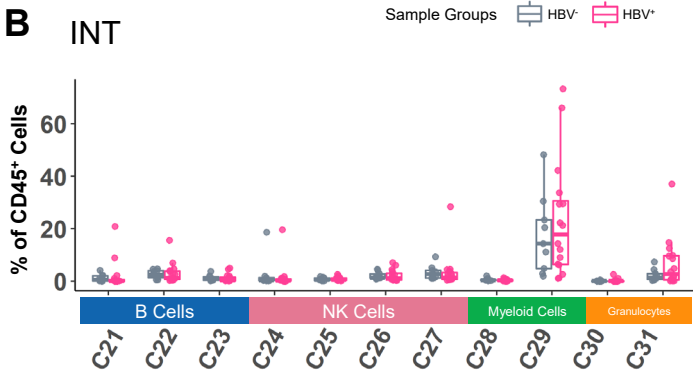

C

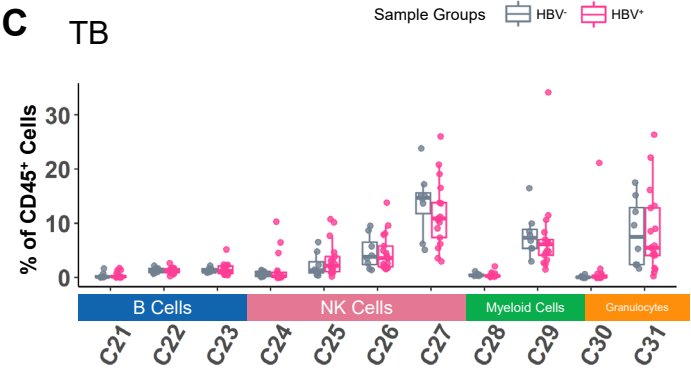

D

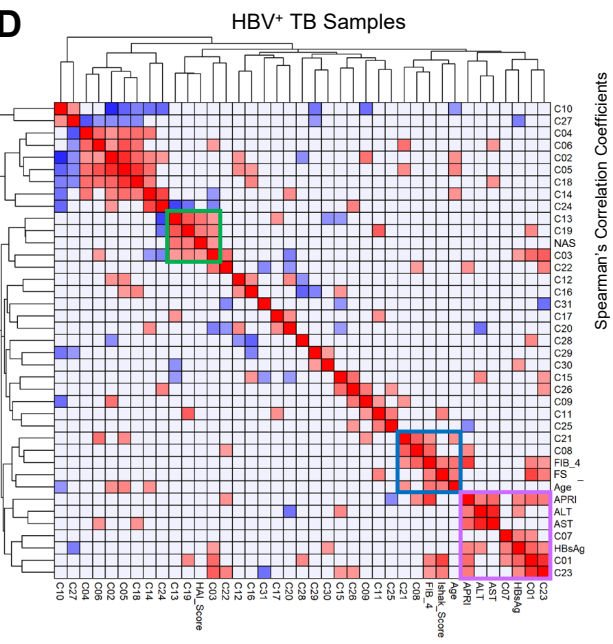

E

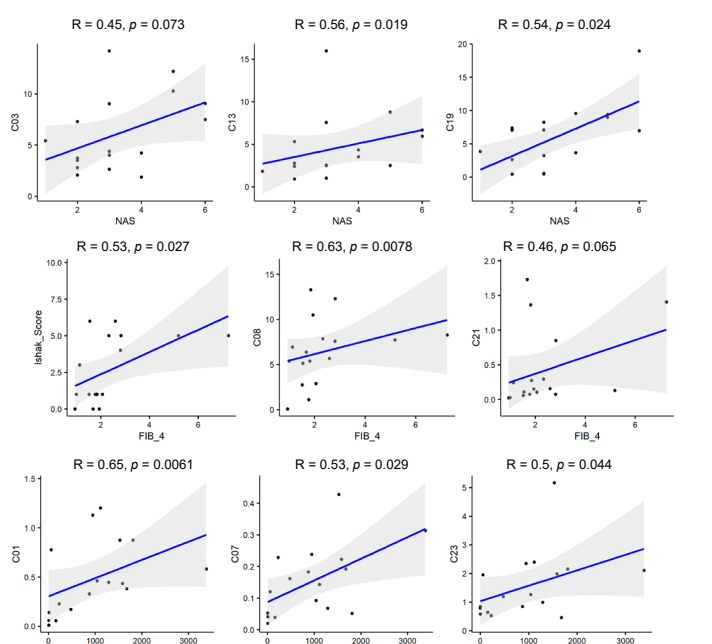

Supplement: Supplementary file 10 — Additional file 10: FigureS3.Correlation of HBV clinical features with immune features. (A) Comparisons ofthe frequencies of major immune subsets across HBV infection and HCC tissues.(B and C) Comparisons of the frequencies of selected meta-clusters between HBV+and HBV- samples in INT (B) and TB (C) tissues. (D) The heatmap of the Spearmancorrelation coefficients between HBV clinical features and immune features inTB tissues, colored by correlation coefficients and for pairs with p-value morethan 0.1 were set to zero. E, The correlation plots between selected HBVclinical features and immune features as in (D). Two-way ANOVA test was used in(A), with *p < 0.05, **p < 0.01, and ***p < 0.001. [file 13046_2023_2710_MOESM10_ESM.pdf]

Figure S4

A

DAPI

PD-1

CD103

CD8

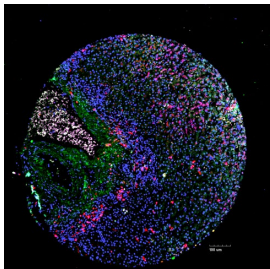

HBV<sup>+</sup> INT

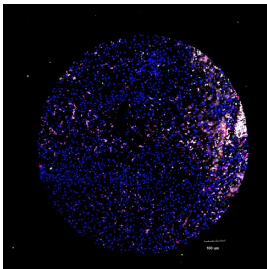

HBV<sup>+</sup> TB

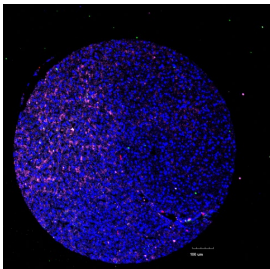

HBV<sup>-</sup> INT

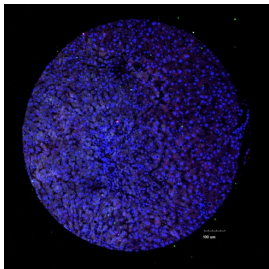

HBV<sup>-</sup> TB

Supplement: Supplementary file 11 — Additional file 11: FigureS4.Validation of the existence of PD-1+CD8+ TRMcells in HBV+ samples. (A) Multiplex immunofluorescence staining offormalin-fixed paraffin-embedded INT and TB tissues of enrolled HCC patients as shown in Figure 4A. [file 13046_2023_2710_MOESM11_ESM.pdf]

Figure S5

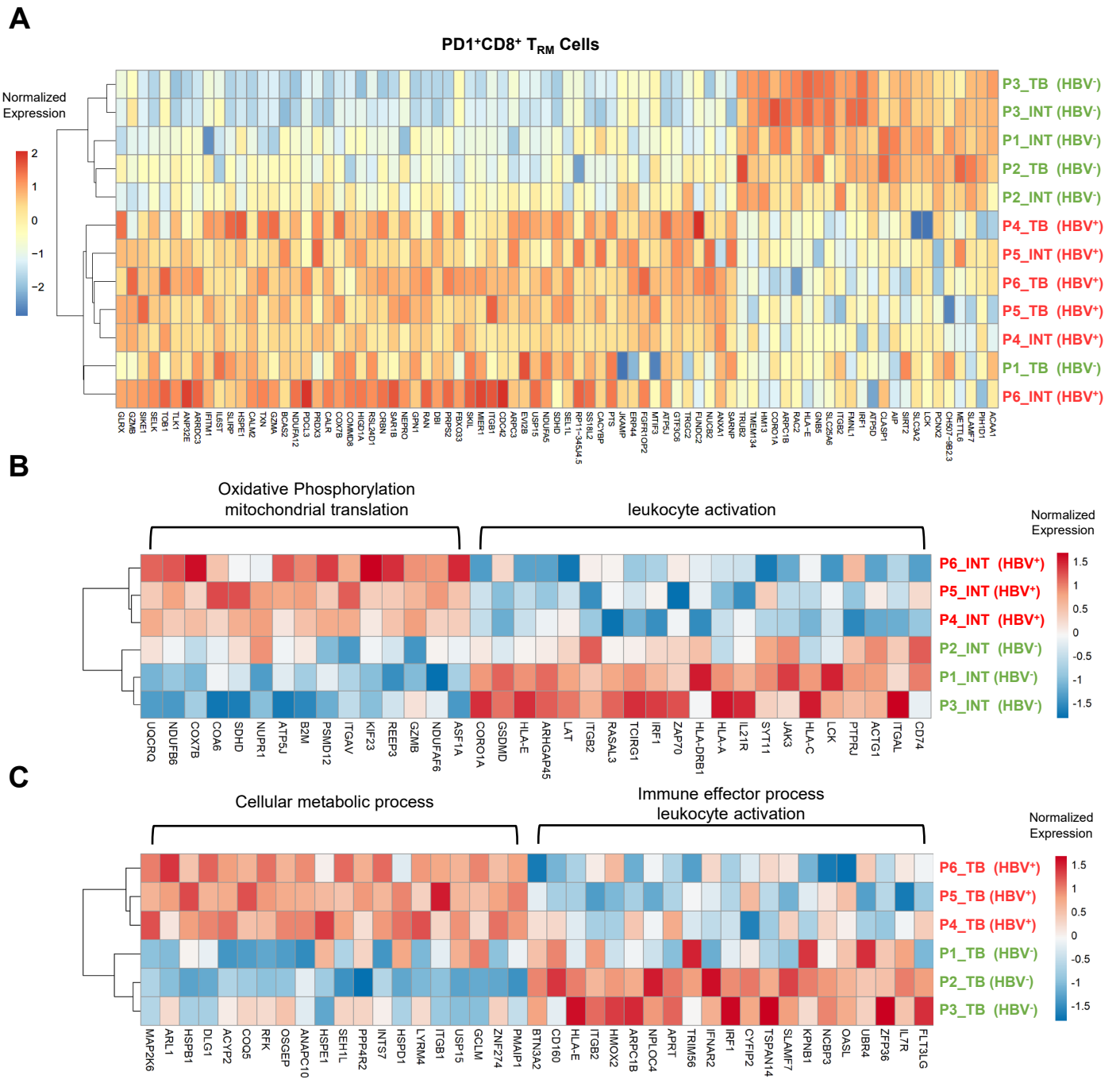

Supplement: Supplementary file 12 — Additional file 12: Figure S5. The distinct geneexpression of PD-1+CD8+ TRM cells across HBVinfection and tissue sites. (A) The heatmap of the normalized expressions ofshared upregulated and downregulated genes between HBV+ and HBV-samples in INT and TB tissues, color-labeled by HBV infection. (B and C) Theheatmap of the normalized expressions of selected functional genes of enrichedGO pathways between HBV+ and HBV- samples in INT tissues(B) and TB tissues (C), color-labeled by HBV infections. [file 13046_2023_2710_MOESM12_ESM.pdf]
