# Supplementary material for: Single-cell analysis reveals HBV-specific PD-1+CD8+ TRM cells in tumor borders are associated with HBV-related hepatic damage and fibrosis in HCC patients
Source: J Exp Clin Cancer Res. 2023 Jun 23;42:152. doi: 10.1186/s13046-023-02710-4 (PMC10288678; doi:10.1186/s13046-023-02710-4)

Figure S6

A

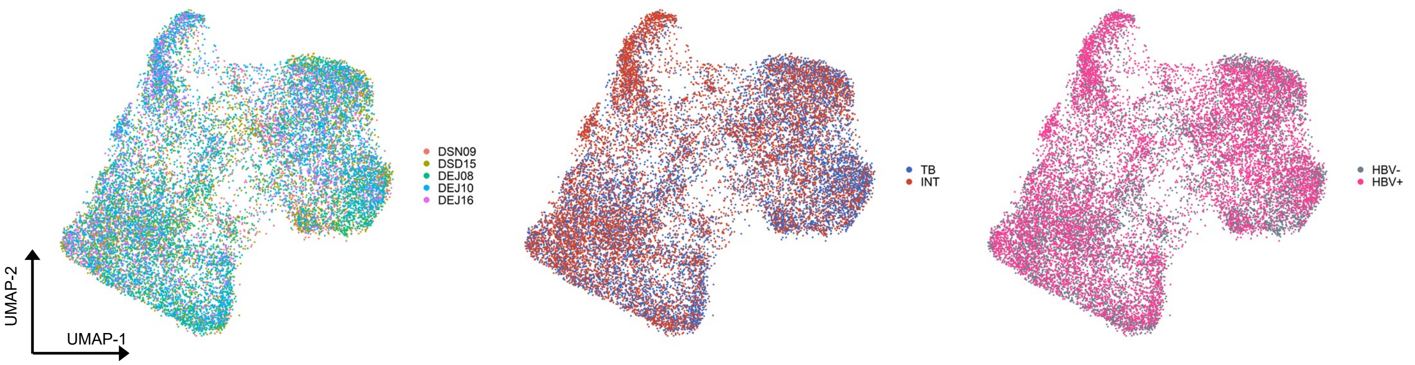

B

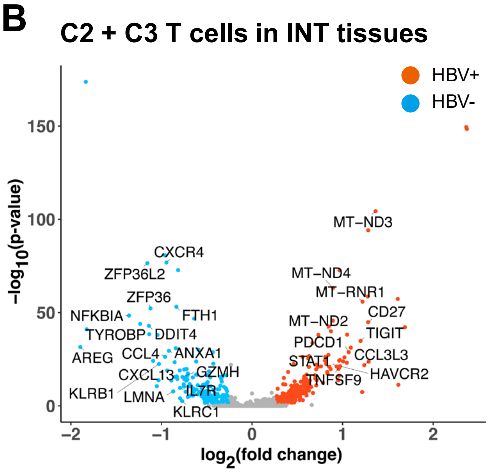

C

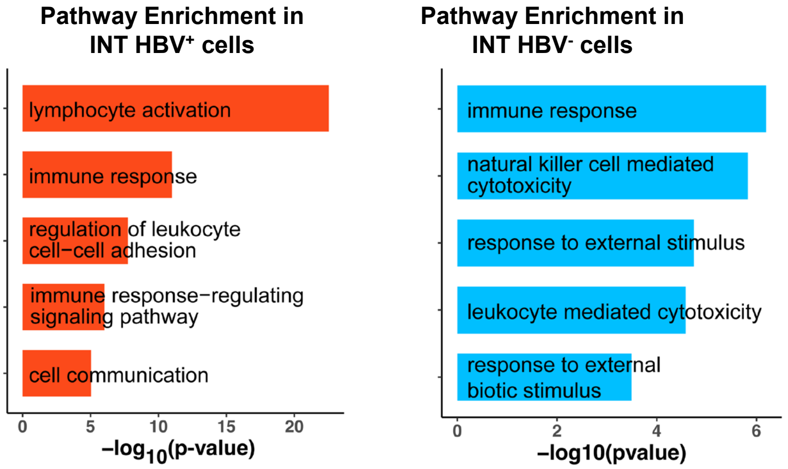

Supplement: Supplementary file 13 — Additional file 13: FigureS6. Analysis results ofscRNA-seq datasets of HCC T cells. (A) The UMAP plots of T cells in HCCpatients as in (Figure 6A), colored by patient ID (left), tissues (middle), andHBV infection status (right). (B)The volcano plots of differentially expressed genes of C2+C3 T cells between HBV+and HBV- samples in INTtissues, labeled with typical marker genes. (C) The GO pathway analyses of shared upregulatedgenes (left) and downregulated genes (right) between HBV+ and HBV-C2+C3 T cells in INT tissues, the pathway nameswere labeled. [file 13046_2023_2710_MOESM13_ESM.pdf]
